# Germline genetic variants were interactively associated with somatic alterations in gastric cancer

**DOI:** 10.1002/cam4.1612

**Published:** 2018-06-20

**Authors:** Xu Zhang, Yuzhuo Wang, Tian Tian, Gangqiao Zhou, Guangfu Jin

**Affiliations:** ^1^ Department of Epidemiology School of Public Health Nanjing Medical University Nanjing China; ^2^ Jiangsu Key Lab of Cancer Biomarkers, Prevention and Treatment Collaborative Innovation Center of Cancer Medicine Nanjing Medical University Nanjing China; ^3^ Department of Epidemiology and Biostatistics School of Public Health Nantong University Nantong China; ^4^ State Key Laboratory of Proteomics Beijing Proteome Research Center Beijing Institute of Radiation Medicine Beijing China; ^5^ National Engineering Research Center for Protein Drugs Beijing China; ^6^ National Center for Protein Sciences at Beijing Beijing China

**Keywords:** association studies, enrichment analysis, gastric cancer, germline variants, somatic alteration

## Abstract

Genome‐wide association studies have identified several germline variants in gastric cancer. Meanwhile, sequencing studies have characterized extensive somatic alterations that arise during gastric carcinogenesis. However, the relationship between the germline variants and somatic alterations is still unclear in gastric cancer. A total of 11 susceptibility loci and 276 driver genes of gastric cancer were determined based on previous studies and publicly available database. An enrichment analysis was made to detect whether driver genes were enriched in susceptibility regions. Besides, we performed a pathway enrichment analysis to find common‐enrich pathways of cancer driver genes and susceptibility genes. Finally, on the basis of the gastric cancer samples and data from TCGA STAD project, we evaluated the associations between susceptibility loci and somatic alterations. Enrichment analysis showed that gastric cancer susceptibility genes were more likely to be enriched in driver genes than in all the genes (*P *= .05). The susceptibility genes and driver genes were commonly enriched in 8 biological pathways. Gastric cancer susceptibility locus of rs2285947 was associated with truncation mutation within Signaling by PDGF pathway (OR = 0.26, 95%CI: 0.12‐0.55, *P *=* *3.93 × 10^−4^). The rs1679709 was connected with COSMIC Signature15 (*P *=* *.026). Moreover, rs1679709 was also associated with copy number values of *RFC4* which is related to Signature15. These results provide evidence for the relationship between germline variants and somatic alterations, which facilitate understanding the interactive mechanism of germline variations with somatic alterations in gastric cancer development.

## 1 INTRODUCTION

Gastric cancer is the second most common cancer and the second leading cause of cancer death in China.^1^ The environmental risk factors for gastric cancer include high‐salt diet, smoking, and infectious agents.^2^ Besides, there are still numerous genetic factors which determine an individual's predisposition to gastric cancer.^3^ Many genetic variations, most of which are single nucleotide polymorphisms (SNPs), have been detected over the past years by the genome‐wide association studies (GWAS) of gastric cancer.^4^ These common susceptibility loci included 1q22 (*MUC1*), 3q13.32 (*ZBTB20*), 5p13.1 (*PRKAA1*), 5q14.3 (*lnc‐POLR3G‐4*), 6p21.1 (*UNC5CL*), 8q24 (*PSCA*), and 10q23 (*PLCE1*).^5^, ^6^, ^7^, ^8^, ^9^, ^10^

In parallel, a growing number of whole‐exome and whole‐genome sequencing studies have been conducted to define the landscape of somatic mutations in gastric cancer. These studies have identified many driver genes, whose mutations confer selective growth advantage to tumor.^11^ Some of these driver genes are previously known cancer genes (eg, *TP53*,* ARID1A,* and *CDH1*), while the others are new‐found significantly mutated genes in gastric cancer (eg, *MUC6*,* CTNNA2*,* GLI3,* and *RNF43*).^4^, ^12^, ^13^, ^14^, ^15^, ^16^, ^17^, ^18^, ^19^, ^20^, ^21^, ^22^ Moreover, the copy number changes and characteristic mutational signatures also play important roles in gastric cancer development.^4^, ^16^, ^17^, ^18^, ^19^

Recent studies have revealed the associations between germline mutations and somatic alterations in tumor development. According to these studies, a large fraction of cancer predisposition genes can contribute to oncogenesis when they have somatic mutation events in tumors.^23^ The germline *MC1R* status may influence somatic mutation burden in melanoma, and the common germline risk variants are connected with total somatic mutation count in breast cancer.^24^, ^25^ In addition, another study reported that oncoprotein EWSR1‐FLI1 preferentially bound to the risk allele of susceptibility SNP rs79965208 in Ewing sarcoma.^26^ However, the associations between the genetic susceptibility variants and somatic alterations in gastric cancer are still unknown.

**Figure 1:**
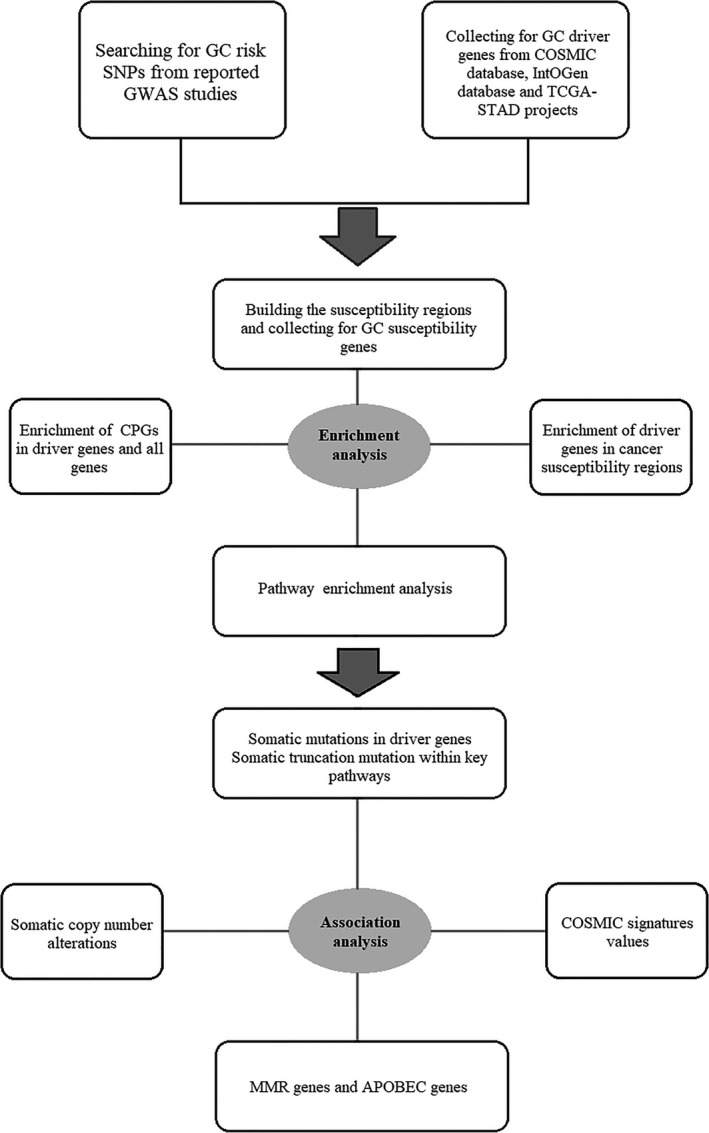
The flowchart of the study design. Firstly, we collected gastric cancer risk SNPs and driver genes. Next we built the susceptibility regions and identified cancer susceptibility genes (CSGs), following by an enrichment analysis to investigate whether driver genes are more likely to locate within susceptibility regions, and whether susceptibility genes and driver genes are enriched in common biological pathways. Finally we made association analyses between gastric cancer risk SNPs genotypes and several somatic events. GC, gastric cancer; CPG, cancer predisposing gene; MMR, DNA mismatch repair pathway

In this study, we set out to examine the association between the established gastric cancer susceptibility loci and the somatic alterations. First, we made enrichment analyses to examine whether driver genes are enriched in germline susceptibility regions, and whether cancer susceptibility genes are enriched in driver genes. Then we made a pathway enrichment analysis to explore whether driver genes and susceptibility genes are enriched in the same pathways. Finally, serial association analyses were conducted to investigate how the risk SNP genotypes affect the somatic alterations during gastric cancer development (Figure 1).

## 2 MATERIALS AND METHODS

### 2.1 Risk SNPs and cancer susceptibility genes

Gastric cancer risk SNPs were extracted from the original gastric cancer GWAS studies in GWAS Catalog.^5^, ^6^, ^7^, ^8^, ^9^, ^10^, ^27^ Only 1 SNP was remained when multiple variants were in linkage disequilibrium (LD, *r* ^2^ ≥ .8), and the minor‐allele frequency (MAF) should be greater or equal than .05. In addition, we included SNP rs1679709 which was reported in a new gastric cancer GWAS study in 2017 and meet the above selection criteria.^28^

Gastric cancer susceptibility regions were defined as 200 kb upstream and downstream of a risk SNP. The protein coding genes located in these susceptibility regions were defined as cancer susceptibility genes (CSGs). Moreover, based on the Genotype‐Tissue Expression (GTEx) v6p database, the protein coding genes whose expression in stomach tissue was associated with risk SNPs or SNPs in high LD (*r* ^2^ ≥ .8) with risk SNPs were also defined as CSGs.^29^ Genomic coordinates and gene symbols of the protein coding genes were gained from GENCODE version 19.^30^

### 2.2 Driver genes

Gastric cancer driver genes were obtained from 3 sources: (1) 16 gastric cancer‐related driver genes from Catalogue of Somatic Mutations in Cancer (COSMIC) Cancer Gene Census(v78)^31^; (2) 175 driver genes of gastric cancer from the Integrative Onco Genomics (IntOGen) database^32^; (3) 108 significantly mutated genes (SMGs) and 26 somatic copy number alteration genes from previously published Whole Genome Sequencing (WGS) or Whole Exome Sequencing (WES) articles.^4^, ^12^, ^13^, ^14^, ^15^, ^16^, ^17^, ^18^, ^19^, ^20^, ^21^, ^22^

### 2.3 Region enrichment analysis and gene‐based enrichment analysis

First of all, we calculated the proportion of driver genes in susceptibility regions. A random sampling of SNP sets was carried out to appraise the background distribution of driver genes, and a total of 10 000 sets of randomly sampled SNPs, which were similar with risk SNP sets in several genomic features, were obtained using the SNPsnap online server. The SNPsnap Web server provide matched sets of SNPs based on allele frequency, number of SNPs in LD, distance to nearest gene and gene density that can be used to calibrate background expectations.^33^ We calculated the number of random SNP sets with proportions of driver genes equal to or larger than that of risk SNPs. Then we divided the calculated number by 10 000 and defined it as the *P* values for enrichment of driver genes in susceptibility regions. To detect whether the length of flanking regions will affect the enrichment result, we defined 50kb, 100kb, 200kb and 500kb upstream and downstream of a risk SNP as the susceptibility regions, calculating *P* values for each susceptibility region separately.

In the gene‐based enrichment analysis, we computed the enrichment ratio of CSGs in all genes (56 318 genes from GENCODE V19) and that in driver genes. The fold enrichment ratio between these 2 ratios was counted as well. The *P* value of fold enrichment ratio was counted using the “phyper” function in R 3.3.1.

### 2.4 Pathway enrichment analysis

Reactome pathways were downloaded from the MSigDB website (http://software.broadinstitute.org/gsea/index.jsp/). Each Reactome pathway included in our analysis should contain at least 1 gastric cancer driver gene or susceptibility gene. We used the “phyper” function as implemented in R to compute the *P* value for enrichment of CSGs or driver genes in a given pathway. The *P* value was adjusted to account for multiple hypotheses testing with Benjamini‐Hochberg correction and we defined a pathway to be significant if the false discovery rate (FDR) ≤ 0.1.

### 2.5 Genotype

We used data from TCGA STAD project to perform association analyses. The germline genotypes were generated using the Affymetrix Genome‐Wide Human SNP Array 6.0. There were 442 cases left after the standard quality control process. Then we conducted genotype imputation using SHAPIT for prephasing and IMPUTE2 for imputation, based on the 1000 Genomes Project Phase III integrated variant set release.^34^, ^35^ Risk SNPs included in the association analysis should meet the standards as follows: imputation info ≥0.5, minor allele frequency (MAF) ≥0.01, and Hardy‐Weinberg equilibrium *P*‐values ≥.001.

### 2.6 Association analysis on somatic mutations within driver genes and somatic truncation mutations within key pathways

The mutation information of gastric cancer was available online, based on the whole‐exome sequencing data supplied by TCGA‐STAD project and we used the mutation annotation file (TCGA.STAD.mutect.a88b4065‐34b4‐4858‐9c16‐55def79c38f2.DR‐6.0.public.maf) from Genomic data commons (GDC) data portal (https://portal.gdc.cancer.gov/projects/TCGA-STAD). The 276 driver genes described above were included into analysis, and for each patient, a driver gene was considered mutated if one or more DNA mutations mapped to this gene.

Pathways where driver genes and CSGs were both significantly enriched in were defined as key pathways. As a result, a total of 8 Reactome pathways were included in our analysis. For each patient, a key pathway was considered to be mutated if one or more truncation mutations were detected in this pathway.

### 2.7 Association analysis on somatic copy number alterations

Output files of SNP6 copy number analysis (GISTIC2) were obtained from the Broad Institute Genome Data Analysis Center (GDAC) Firehose portal (http://gdac.broadinstitute.org/runs/analyses__latest/reports/cancer/STAD-TP/CopyNumber_Gistic2/nozzle.html). We extracted the CNV regional information from the “all_lesions.conf_99.txt” file, and it contains data about the significant regions of amplification and deletion as well as which samples are amplified or deleted in each of these regions. Besides, we extracted gene‐level copy number values of gastric cancer driver genes in each TCGA STAD sample from the “all_data_by_genes.txt” file. We take the absolute values of the copy number values and our analysis only focus on the copy number values of 276 driver genes.

### 2.8 Association analysis on COSMIC signatures

We calculated the weight of each mutational signature contributing to an individual TCGA STAD sample using the “deconstructSigs” package in R 3.3.1 (package details in https://github.com/raerose01/deconstructSigs) with the mutation annotation file described above (TCGA.STAD.mutect.a88b4065‐34b4‐4858‐9c16‐55def79c38f2.DR‐6.0.public.maf). We included the 11 COSMIC signatures which had been reported in gastric cancer into our analysis (COSMIC signatures 1, 2, 5, 13, 15, 17, 18, 20, 21, 26, and 28). For each patient, the weights of each mutational signature were used as somatic phenotype.

### 2.9 Collection of DNA mismatch repair genes

DNA mismatch repair (MMR) genes were collected from 2 resources: identified MMR genes in a reported study and the genes in KEGG mismatch repair pathway.^21^ As a result, a total of 26 MMR genes were included in our analysis (Table S1).

### 2.10 Statistical analyses for association analysis

Logistic regression was performed for binary phenotypes, and multiple linear regression was performed for quantitative traits in the association analysis progress. The additive model was utilized in our study, and we controlled for age, gender, clinical stage, and the first 10 principal components. The clinical information (age, gender, and clinical stage) was obtained from GDC data portal, and the missing clinical variable values were imputed with the corresponding median values in our study. The association p values would be adjusted by Benjamini‐Hochberg correction method, and all the tests were two‐sided, a false discovery rate (FDR) of 0.1 was used as significance threshold. All the association analyses were conducted in R‐3.3.1 (http://www.R-project.org/).

## 3 RESULTS

**Figure 2:**
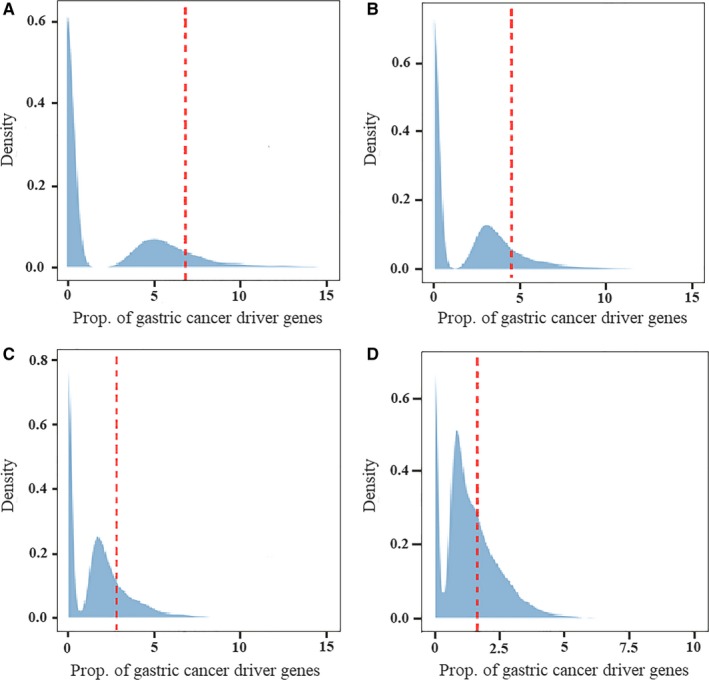
The enrichment analysis of Driver genes in SNP susceptibility regions. The figure showed the proportion results of gastric cancer driver genes in the 4 SNP susceptibility regions: A, The 50 kb‐amplification susceptibility regions. B, The 100 kb‐amplification susceptibility regions. C, The 200 kb‐amplification susceptibility regions. D, The 500 kb‐amplification susceptibility regions

**Figure 3:**
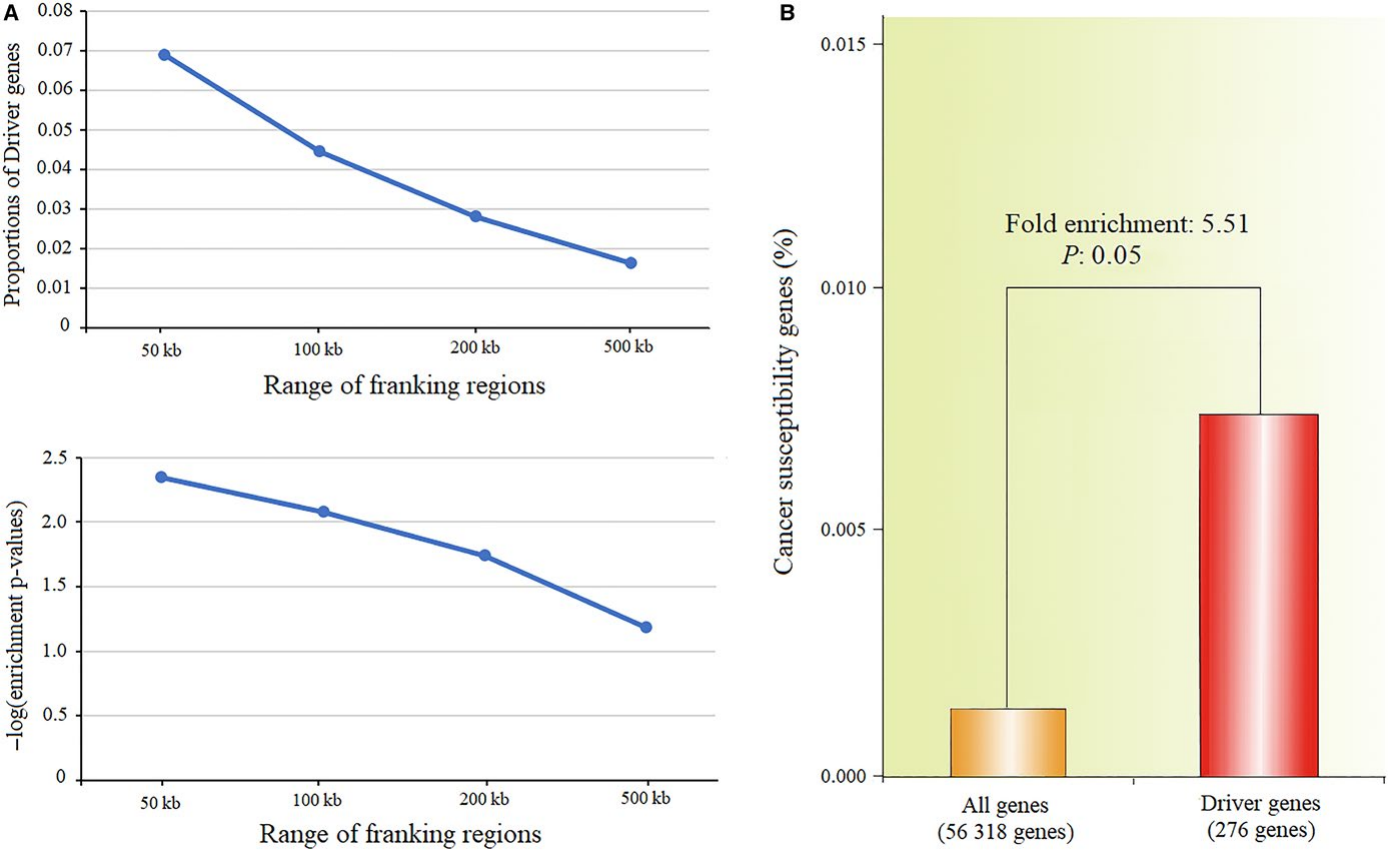
The tendency of Driver genes in SNP susceptibility region and the gene‐based enrichment analysis. A, The trend of proportions of gastric cancer driver genes and −log10 transformed enrichment p‐values of driver genes in 50 kb, 100 kb, 200 kb, and 500 kb upstream and downstream of risk SNPs. B, A bar graph of the percentage of CSGs in the 276 driver genes compared with all genes (56 318) in GENCODE (V19)

We identified 11 gastric cancer risk SNPs according to our definitions and quality control standards (Table S2). Besides, we also identified 74 cancer susceptibility genes and collected 276 driver genes from COSMIC Cancer Gene Census, IntOGen database, and reported WGS or WES studies (Tables S3 and S4). With the enrichment analysis, we found a trend that the proportion of driver genes in susceptibility regions decreased with the increasing size of susceptibility regions, as from 50 kb to 500 kb upstream and downstream of risk SNPs. Inversely, the enrichment *P*‐values increased from 0.0956, 0.1248, 0.175 to 0.3064 (Figures 2 and 3A, Table S5).

**Figure 4:**
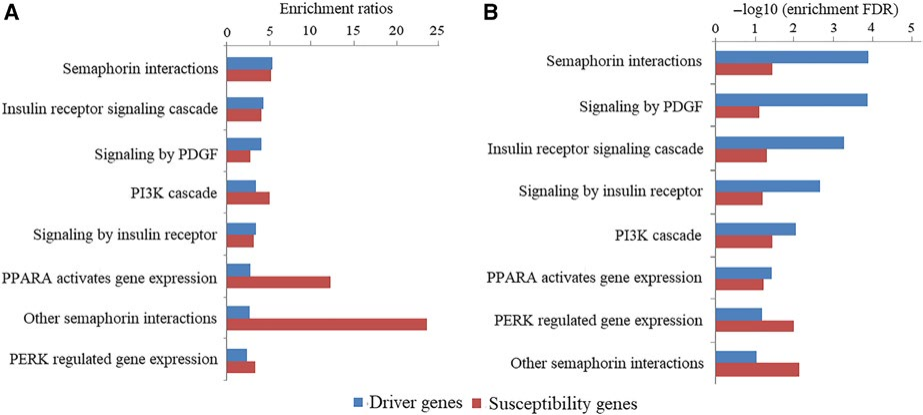
Reactome pathways with FDR<0.1 in hyper‐representative test for gastric cancer driver genes and gastric cancer susceptibility genes. A, Enrichment ratios of significant pathways. B, −log10 transformed false discovery rates (FDRs) of significant pathways

Gene‐based enrichment analysis showed that 2 CSGs were among the 276 driver genes (0.72%), which represented a 5.51‐fold enrichment compared with the 56 318 annotated genes in GENCODE (*P *=* *.05, Figure 3B). According to pathway enrichment analysis result, CSGs and driver genes were commonly enriched in 8 biological pathways. These pathways included insulin receptor signaling cascade, PI3K cascade, PPARA activates gene expression, semaphoring interactions, signaling by insulin receptor, signaling by PDGF, PERK regulated gene expression, and other semaphoring interactions (Figure 4, Table S6).

Association analysis identified a total of 130 associations (*P *<* *.05) between somatic mutations in driver genes and risk SNP genotypes, of which the strongest associations were rs9841504 at 3q13.32 with *POLE* (*P *=* *7.36 × 10^−4^, Table S7) and rs2285947 at 7p15.3 with *SOS1* (*P *=* *2.06 × 10^−3^, Table S7). However, no association remained significant after FDR adjustment (Table S7). We wondered whether the 130 SNP‐gene pairs remain significant on gene expression level, followed with an expression quantitative trait loci (eQTL) analysis in stomach tissues based on GTEx. As a result, the eQTL analysis identified 11 associations between the gene expression levels and genotypes (*P *<* *.05, Figure S1, Table S8). However, no results remain significant after FDR adjustment.

Analysis of truncation mutations in key pathways identified one significant result with FDR < 0.1 (Table S9). The risk allele (A) of rs2285947 (7p15.3) was associated with Signaling by PDGF pathway (OR = 0.26, 95% CI: 0.12‐0.55, *P *=* *3.93 × 10^−4^) (Table 1). To further explore the association between rs2285947 and signaling by PDGF pathway, we performed a stratified analysis by age, sex, ethnicity, and tumor stage status. As shown in Table S10, the association remained significant in both age groups, females and Caucasians.

**Table 1:** The associations between rs2285947 and truncation mutation within Signaling by PDGF pathway

Genotypes	Cases with mutation (n = 28)^a^		Cases without mutation (n = 386)^a^		OR (95% CI)^b^	*P* value^b^
	N	%	N	%		
GG	18	64.29	131	33.94	1	
GA	10	35.71	191	49.48	0.36 (0.15‐0.85)	1.94E−02
AA	0	0	64	16.58	‐	‐
GA/AA	10	35.71	255	66.06	0.24 (0.10‐0.58)	1.38E−03
Additive model					0.26 (0.12‐0.55)	3.93E−04

a Patients with or without somatic truncation mutations in pathway.

b Odds ratios, 95% confidence intervals, and *P* values were calculated using logistic regression models adjusting for age, gender, clinical stage, and the first 10 principal components.

We investigated the interactions between risk SNP genotypes and somatic copy number alterations (SCNAs). Results showed there were several “germline‐SCNAs” associations, like rs2494938 (6p21.1) with the *ERBB3* gene copy number values (*P *=* *2.38 × 10^−3^), rs13361707 (5p13.1) with the significant focal amplification region 12p12.1 (*P *=* *9.03 × 10^−3^), and rs1679709 (6p22.1) with the significant focal deletion region 9q21.11 (*P *=* *5.33 × 10^−3^) (Tables S11‐S13). However, we did not find any association with FDR < 0.1.

**Figure 5:**
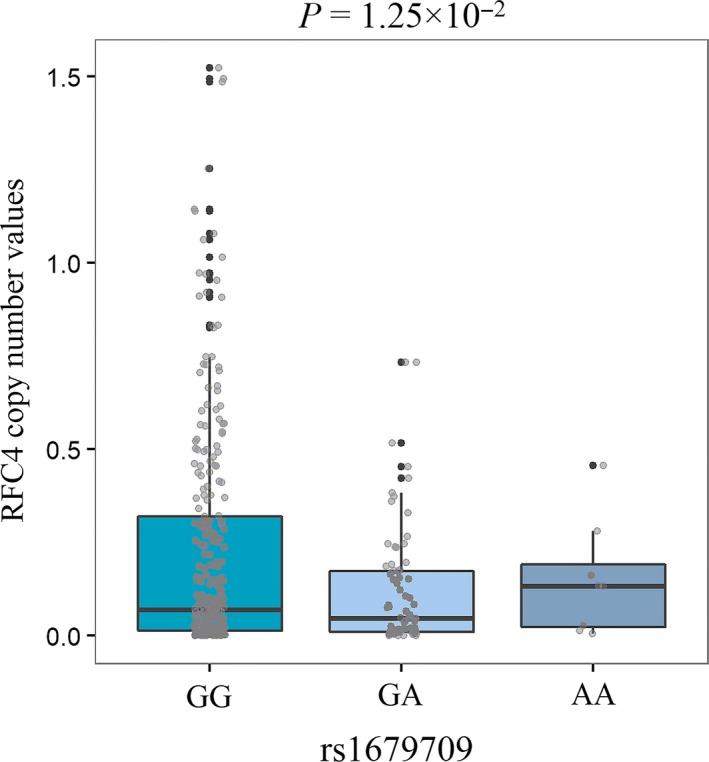
The association between genotypes of rs1679709 and gene copy number values of RFC4 gene. The box plot displays the first and third quartiles (top and bottom of the boxes), the median (band inside the boxes), and the lowest and highest point within 1.5 times the interquartile range of the lower and higher quartile

There were 11 patterns of mutational signatures found in gastric cancer, and our analysis identified 5 pairs of associations with *P*‐values less than .05 between risk SNPs genotypes and the weight of mutational signature (Table S14). Signature15 were associated with rs1679709 (6p22.1) (*P* = .026), as signature15 was DNA mismatch repair related signature according to COSMIC website. Thus, we further investigated whether rs1679709 genotypes were associated with expression of MMR genes. The results showed rs1679709 was concerned with 1 gene in gene‐level copy number values: *RFC4* (*P *=* *1.25 × 10^−2^) (Figure 5, Table S15).

## 4 DISCUSSION

Region enrichment analysis showed that there was no difference between the proportion of driver genes in cancer susceptibility regions and in the background regions. A recent study has reported that cancer susceptibility regions have gene mutation frequencies comparable to background mutation frequencies.^36^ Besides, with the gene‐based enrichment analysis we observed that gastric cancer CSGs were more likely to be enriched in driver genes, although it was only marginal significant. It makes sense as somatic cancer driver mutations and germline cancer predisposing mutations are highly overlapped, whereas such mutual interrogation had been underestimated due to the artifact of different research approaches.^23^ In addition, pathway enrichment analysis found CSGs and driver genes were commonly enriched in 8 Reactome pathways. The result implied that germline mutations and somatic mutations may work together in some particular biological pathways during gastric cancer development.

Our interaction analysis of somatic mutations in driver genes and risk SNPs genotypes identified several significant SNP‐gene pairs. As a previous study reported, genetic background could influence the somatic evolution of a tumor by modifying the likelihood of acquiring mutations in specific cancer genes,^37^ which could possibly explain why there were a number of SNP‐gene pairs found in our study.

A pathway‐based analysis was performed later, and we detected that the risk allele of rs2285947 (A) was significantly associated with the occurrence of truncation mutations in Reactome pathway Signaling by PDGF. PDGF pathway has long been implicated in cancers and is known to be involved in many biological processes.^38^ Rs2285947 is an intronic variant in *DNAH11* and is able to regulate expression levels of *DNAH11* in stomach tissues based on GTEx (*P *=* *3.40 × 10^−5^), as *DNAH11* is a microtubule‐dependent motor ATPase according to GeneCards website. Results showed that risk allele of rs2285947 (A) has an inverse association with *DNAH11* gene expression (effect size = −0.28) and PDGF signaling pathway (Beta = −1.35) at the same time. One possible interpretation is that rs2285947 may affect the ATPase activity by regulating *DNAH11* expression level, and it may influence the release of PDGF‐R, since E5 oncoprotein can form a ternary complex with PDGF‐R and the 16K subunit of the vacuolar V‐ATPase.^39^ These PDGF receptors may dimerize and undergo autophosphorylation and then attract downstream effectors to transduct the signal into the cell.^40^, ^41^, ^42^ According to the “two‐hit” model which explain the interaction of germline and somatic mutation, individuals with elevated genetic predisposition may require fewer stages to develop a tumor than those at lower genetic risk. Thus, it is possible that the observed inverse association between rs2285947 and PDGF signaling pathway is a result of the continuous process of cancer development, where both germline variants and somatic alterations contribute to the development of gastric cancer.

In the past few years, there are 30 patterns of mutational signatures be found across the spectrum of human cancer types from many large‐scale analyses,^43^, ^44^, ^45^, ^46^, ^47^ including 11 types of signatures found in gastric cancer. We observed a significant association between rs1679709 and the weights of COSMIC Signature 15. Signature 15 is linked to defective DNA mismatch repair according to COSMIC website.^48^ In the following analysis, we found gene copy number values of MMR gene *RFC4* was also related to rs1679709 genotypes. Therefore, rs1679709 might influence defective DNA mismatch repair associated mutational signatures in gastric cancer, via regulating the copy number changes in relevant genes like RFC4.

Nowadays fundamental gaps remain in our knowledge of how normal cells evolve into cancer cells and what are the vital potential genetic backgrounds. In this research, we did identified several interactions between germline susceptibility loci and somatic mutations in gastric cancer. However, the exact mechanism how the germline alleles affect the progression of later somatic events is still unknown because of the limit of study sample scale and the lack of functional experiments. In addition, it is a limitation that there are only 89 Asians among 443 cases in TCGA STAD, but most germline susceptibility loci were identified in Asian populations. We believe with more susceptibility loci discovered and larger samples of sequencing studies performed in the future, especially in Asian population, the networks of informative interactions between germline mutation and somatic mutation in gastric cancer will eventually be revealed, which may make contributions to the precision medicine.

## CONFLICT OF INTEREST

The authors have no conflict of interest.

## References

[1] Chen W , Zheng R , Baade PD , et al. Cancer statistics in China, 2015. CA Cancer J Clin. 2016;66:115‐132. 26808342 10.3322/caac.21338

[2] Ferlay J , Shin HR , Bray F , Forman D , Mathers C , Parkin DM . Estimates of worldwide burden of cancer in 2008: GLOBOCAN 2008. Int J Cancer. 2010;127:2893‐2917. 21351269 10.1002/ijc.25516

[3] Sampson JN , Wheeler WA , Yeager M , et al. Analysis of heritability and shared heritability based on genome‐wide association studies for thirteen cancer types. J Natl Cancer Inst. 2015;107:djv279. 26464424 10.1093/jnci/djv279 PMC4806328

[4] Tan P , Yeoh KG . Genetics and molecular pathogenesis of gastric adenocarcinoma. Gastroenterology. 2015;149:1153‐1162.e3. 26073375 10.1053/j.gastro.2015.05.059

[5] Saeki N , Saito A , Choi IJ , et al. A functional single nucleotide polymorphism in mucin 1, at chromosome 1q22, determines susceptibility to diffuse‐type gastric cancer. Gastroenterology. 2011;140:892‐902. 21070779 10.1053/j.gastro.2010.10.058

[6] Shi Y , Hu Z , Wu C , et al. A genome‐wide association study identifies new susceptibility loci for non‐cardia gastric cancer at 3q13.31 and 5p13.1. Nat Genet. 2011;43:1215‐1218. 22037551 10.1038/ng.978

[7] Wang Z , Dai J , Hu N , et al. Identification of new susceptibility loci for gastric non‐cardia adenocarcinoma: pooled results from two Chinese genome‐wide association studies. Gut. 2017;66:581‐587. 26701879 10.1136/gutjnl-2015-310612 PMC4963301

[8] Hu N , Wang Z , Song X , et al. Genome‐wide association study of gastric adenocarcinoma in Asia: a comparison of associations between cardia and non‐cardia tumours. Gut. 2016;65:1611‐1618. 26129866 10.1136/gutjnl-2015-309340 PMC5568652

[9] Sakamoto H , Yoshimura K , Saeki N , et al. Genetic variation in PSCA is associated with susceptibility to diffuse‐type gastric cancer. Nat Genet. 2008;40:730‐740. 18488030 10.1038/ng.152

[10] Abnet CC , Freedman ND , Hu N , et al. A shared susceptibility locus in PLCE1 at 10q23 for gastric adenocarcinoma and esophageal squamous cell carcinoma. Nat Genet. 2010;42:764‐767. 20729852 10.1038/ng.649 PMC2947317

[11] Martincorena I , Campbell PJ . Somatic mutation in cancer and normal cells. Science. 2015;349:1483‐1489. 26404825 10.1126/science.aab4082

[12] Wang K , Kan J , Yuen ST , et al. Exome sequencing identifies frequent mutation of ARID1A in molecular subtypes of gastric cancer. Nat Genet. 2011;43:1219‐1223. 22037554 10.1038/ng.982

[13] Nagarajan N , Bertrand D , Hillmer AM , et al. Whole‐genome reconstruction and mutational signatures in gastric cancer. Genome Biol. 2012;13:R115. 23237666 10.1186/gb-2012-13-12-r115 PMC4056366

[14] Zang ZJ , Cutcutache I , Poon SL , et al. Exome sequencing of gastric adenocarcinoma identifies recurrent somatic mutations in cell adhesion and chromatin remodeling genes. Nat Genet. 2012;44:570‐574. 22484628 10.1038/ng.2246

[15] Lawrence MS , Stojanov P , Polak P , et al. Mutational heterogeneity in cancer and the search for new cancer‐associated genes. Nature. 2013;499:214‐218. 23770567 10.1038/nature12213 PMC3919509

[16] Cancer Genome Atlas Research Network . Comprehensive molecular characterization of gastric adenocarcinoma. Nature. 2014;513:202‐209. 25079317 10.1038/nature13480 PMC4170219

[17] Kakiuchi M , Nishizawa T , Ueda H , et al. Recurrent gain‐of‐function mutations of RHOA in diffuse‐type gastric carcinoma. Nat Genet. 2014;46:583‐587. 24816255 10.1038/ng.2984

[18] Wang K , Yuen ST , Xu J , et al. Whole‐genome sequencing and comprehensive molecular profiling identify new driver mutations in gastric cancer. Nat Genet. 2014;46:573‐582. 24816253 10.1038/ng.2983

[19] Chen K , Yang D , Li X , et al. Mutational landscape of gastric adenocarcinoma in Chinese: implications for prognosis and therapy. Proc Natl Acad Sci USA. 2015;112:1107‐1112. 25583476 10.1073/pnas.1422640112 PMC4313862

[20] Cristescu R , Lee J , Nebozhyn M , et al. Molecular analysis of gastric cancer identifies subtypes associated with distinct clinical outcomes. Nat Med. 2015;21:449‐456. 25894828 10.1038/nm.3850

[21] Li X , Wu WK , Xing R , et al. Distinct subtypes of gastric cancer defined by molecular characterization include novel mutational signatures with prognostic capability. Cancer Res. 2016;76:1724‐1732. 26857262 10.1158/0008-5472.CAN-15-2443

[22] Hu N , Kadota M , Liu H , et al. Genomic landscape of somatic alterations in esophageal squamous cell carcinoma and gastric cancer. Cancer Res. 2016;76:1714‐1723. 26857264 10.1158/0008-5472.CAN-15-0338 PMC4873357

[23] Rahman N . Realizing the promise of cancer predisposition genes. Nature. 2014;505:302‐308. 24429628 10.1038/nature12981 PMC4975511

[24] Robles‐Espinoza CD , Roberts ND , Chen S , et al. Germline MC1R status influences somatic mutation burden in melanoma. Nat Commun. 2016;7:12064. 27403562 10.1038/ncomms12064 PMC4945874

[25] Zhu B , Mukherjee A , Machiela MJ , et al. An investigation of the association of genetic susceptibility risk with somatic mutation burden in breast cancer. Br J Cancer. 2016;115:752‐760. 27467053 10.1038/bjc.2016.223 PMC5023771

[26] Gomez NC , Davis IJ . Linking germline and somatic variation in Ewing sarcoma. Nat Genet. 2015;47:964‐965. 26313223 10.1038/ng.3387

[27] Jin G , Ma H , Wu C , et al. Genetic variants at 6p21.1 and 7p15.3 are associated with risk of multiple cancers in Han Chinese. Am J Hum Genet. 2012;91:928‐934. 23103227 10.1016/j.ajhg.2012.09.009 PMC3487121

[28] Zhu M , Yan C , Ren C , et al. Exome array analysis identifies variants in SPOCD1 and BTN3A2 that affect risk for gastric cancer. Gastroenterology. 2017;152:2011‐2021. 28246015 10.1053/j.gastro.2017.02.017

[29] GTEx Consortium . The Genotype‐Tissue Expression (GTEx) project. Nat Genet. 2013;13:307‐308. 10.1038/ng.2653 PMC4010069 23715323

[30] Harrow J , Frankish A , Gonzalez JM , et al. GENCODE: the reference human genome annotation for The ENCODE Project. Genome Res. 2012;22:1760‐1774. 22955987 10.1101/gr.135350.111 PMC3431492

[31] Futreal PA , Coin L , Marshall M , et al. A census of human cancer genes. Nat Rev Cancer. 2004;4:177‐183. 14993899 10.1038/nrc1299 PMC2665285

[32] Gonzalez‐Perez A , Perez‐Llamas C , Deu‐Pons J , et al. IntOGen‐mutations identifies cancer drivers across tumor types. Nat Methods. 2013;10:1081‐1082. 24037244 10.1038/nmeth.2642 PMC5758042

[33] Pers TH , Timshel P , Hirschhorn JN . SNPsnap: a Web‐based tool for identification and annotation of matched SNPs. Bioinformatics. 2015;31:418‐420. 25316677 10.1093/bioinformatics/btu655 PMC4308663

[34] Delaneau O , Marchini J , Zagury JF . A linear complexity phasing method for thousands of genomes. Nat Methods. 2011;9:179‐181. 22138821 10.1038/nmeth.1785

[35] Howie BN , Donnelly P , Marchini J . A flexible and accurate genotype imputation method for the next generation of genome‐wide association studies. PLoS Genet. 2009;5:e1000529. 19543373 10.1371/journal.pgen.1000529 PMC2689936

[36] Machiela MJ , Ho BM , Fisher VA , Hua X , Chanock SJ . Limited evidence that cancer susceptibility regions are preferential targets for somatic mutation. Genome Biol. 2015;16:193. 26374197 10.1186/s13059-015-0755-5 PMC4571124

[37] Carter H , Marty R , Hofree M , et al. Interaction landscape of inherited polymorphisms with somatic events in cancer. Cancer Discov. 2017;7:410‐423. 28188128 10.1158/2159-8290.CD-16-1045 PMC5460679

[38] Shih AH , Holland EC . Platelet‐derived growth factor (PDGF) and glial tumorigenesis. Cancer Lett. 2006;232:139‐147. 16139423 10.1016/j.canlet.2005.02.002

[39] Sparkowski J , Anders J , Schlegel R . E5 oncoprotein retained in the endoplasmic reticulum/cis Golgi still induces PDGF receptor autophosphorylation but does not transform cells. EMBO J. 1995;14:3055‐3063. 7621820 10.1002/j.1460-2075.1995.tb07308.x PMC394366

[40] Heldin CH , Ostman A , Ronnstrand L . Signal transduction via platelet‐derived growth factor receptors. Biochim Biophys Acta. 1998;1378:F79‐F113. 9739761 10.1016/s0304-419x(98)00015-8

[41] Heldin CH , Westermark B . Mechanism of action and in vivo role of platelet‐derived growth factor. Physiol Rev. 1999;79:1283‐1316. 10508235 10.1152/physrev.1999.79.4.1283

[42] Fredriksson L , Li H , Eriksson U . The PDGF family: four gene products form five dimeric isoforms. Cytokine Growth Factor Rev. 2004;15:197‐204. 15207811 10.1016/j.cytogfr.2004.03.007

[43] Nik‐Zainal S , Alexandrov LB , Wedge DC , et al. Mutational processes molding the genomes of 21 breast cancers. Cell. 2012;149:979‐993. 22608084 10.1016/j.cell.2012.04.024 PMC3414841

[44] Alexandrov LB , Nik‐Zainal S , Wedge DC , Campbell PJ , Stratton MR . Deciphering signatures of mutational processes operative in human cancer. Cell Rep. 2013;3:246‐259. 23318258 10.1016/j.celrep.2012.12.008 PMC3588146

[45] Alexandrov LB , Nik‐Zainal S , Wedge DC , et al. Signatures of mutational processes in human cancer. Nature. 2013;500:415‐421. 23945592 10.1038/nature12477 PMC3776390

[46] Helleday T , Eshtad S , Nik‐Zainal S . Mechanisms underlying mutational signatures in human cancers. Nat Rev Genet. 2014;15:585‐598. 24981601 10.1038/nrg3729 PMC6044419

[47] Alexandrov LB , Stratton MR . Mutational signatures: the patterns of somatic mutations hidden in cancer genomes. Curr Opin Genet Dev. 2014;24:52‐60. 24657537 10.1016/j.gde.2013.11.014 PMC3990474

[48] Dietlein F , Thelen L , Reinhardt HC . Cancer‐specific defects in DNA repair pathways as targets for personalized therapeutic approaches. Trends Genet. 2014;30:326‐339. 25017190 10.1016/j.tig.2014.06.003

