# Supplementary material for: Germline genetic variants were interactively associated with somatic alterations in gastric cancer
Source: Cancer Med. 2018 Jun 20;7(8):3912–20. doi: 10.1002/cam4.1612 (PMC6089170; doi:10.1002/cam4.1612)
Supplement: Supplementary file 1 [file CAM4-7-3912-s001.pdf]

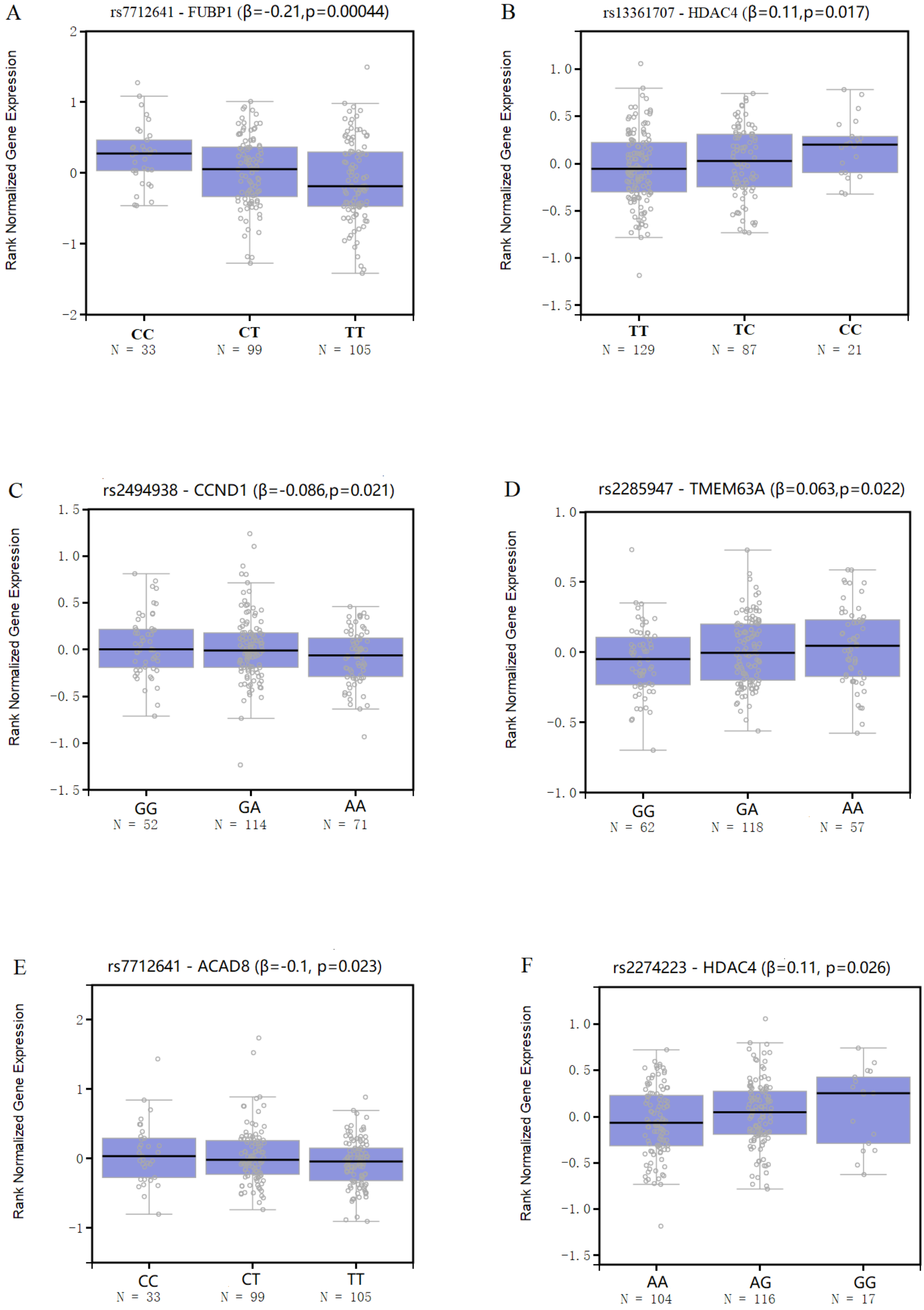
A
rs7712641 - FUBP1 (β=-0.21,p=0.00044)
Rank Normalized Gene Expression
2
1
0
−1
−2
CC
N = 33
CT
N = 99
TT
N = 105
B
rs13361707 - HDAC4 (β=0.11,p=0.017)
Rank Normalized Gene Expression
1.0
0.5
0.0
−0.5
−1.0
−1.5
TT
N = 129
TC
N = 87
CC
N = 21
C
rs2494938 - CCND1 (β=-0.086,p=0.021)
Rank Normalized Gene Expression
1.5
1.0
0.5
0.0
−0.5
−1.0
−1.5
GG
N = 52
GA
N = 114
AA
N = 71
D
rs2285947 - TMEM63A (β=0.063,p=0.022)
Rank Normalized Gene Expression
1.0
0.5
0.0
−0.5
−1.0
GG
N = 62
GA
N = 118
AA
N = 57
E
rs7712641 - ACAD8 (β=-0.1, p=0.023)
Rank Normalized Gene Expression
2
1
0
−1
CC
N = 33
CT
N = 99
TT
N = 105
F
rs2274223 - HDAC4 (β=0.11, p=0.026)
Rank Normalized Gene Expression
1.0
0.5
0.0
−0.5
−1.0
−1.5
AA
N = 104
AG
N = 116
GG
N = 17

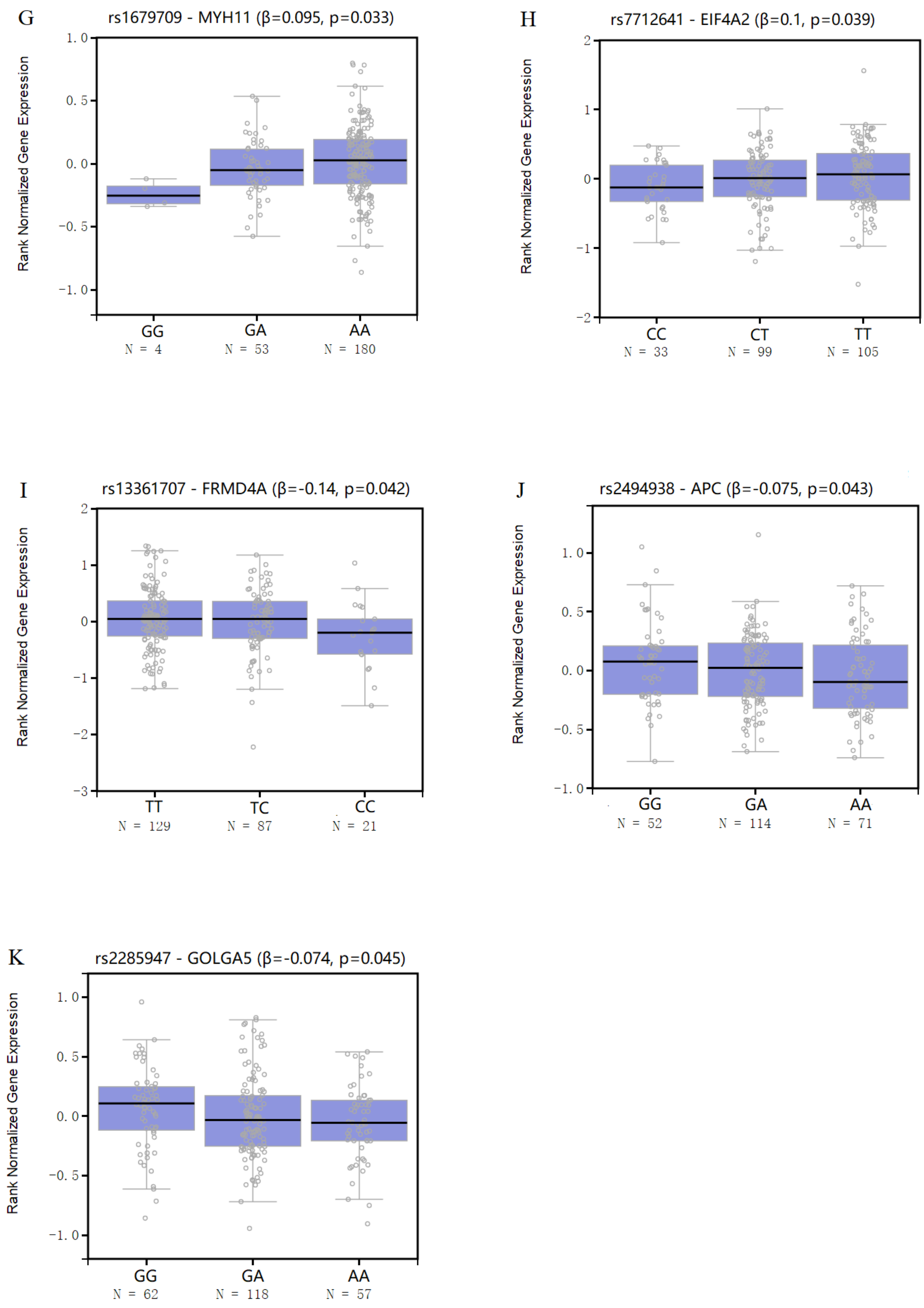

Figure S1. The boxplot shows the 11 pairs of associations between risk SNPs and driver

gene expression in stomach tissues based on GTEx project. The expression of genes was normalized. The box plot displays the first and third quartiles, the median, and the lowest and highest point within 1.5 times the interquartile range of the lower and higher
